# Predictors of happiness during the COVID-19 pandemic in mothers of infants and/or preschoolers: a pre-COVID-19 comparative study in Japan

**DOI:** 10.1265/ehpm.22-00008

**Published:** 2022-03-26

**Authors:** Miyako Kimura, Kazushige Ide, Kazuki Kimura, Toshiyuki Ojima

**Affiliations:** 1 Department of Preventive Medicine, St. Marianna University School of Medicine, 2-16-1, Sugao, Miyamae-ku, Kawasaki-shi, 216-8511, Japan; 2 Department of Social Preventive Medical Sciences, Center for Preventive Medical Sciences, Chiba University, Inage-ku, Chiba, Japan; 3 Graduate School of Applied Life Science, Nippon Veterinary and Life Science University, Musashino-shi, Tokyo, Japan; 4 Hamamatsu University School of Medicine, Community Health & Preventive Medicine, Hamamatsu city, Shizuoka, Japan

**Keywords:** Coping, COVID-19, Happiness, Maternal and child health, Positive attitudes, Positive thinking, Preventive behavior, Psychological well-being, Satisfaction

## Abstract

**Background:**

Happiness may help to prevent negative physiological outcomes in response to life events; however, factors contributing to happiness during the COVID-19 pandemic have not been longitudinally investigated. This study explored the predictors of happiness in mothers of young children in Japan using comparable data that were obtained before and during the COVID-19 pandemic.

**Methods:**

We conducted the baseline survey in February 2020, and 4 months later, we also conducted the follow-up survey. Throughout all 47 prefectures in Japan, 4,700 (100 respondents/prefecture) mothers of infants and/or preschoolers (0–6 years) participated in the baseline online survey; 2,489 of these also participated in the follow-up survey.

**Results:**

We performed hierarchical multiple regression analysis and our final model indicated that maternal happiness during COVID-19 pandemic was positively related to employment status (homemaker, β = 0.052, p = 0.014), levels of available social support (average, β = 0.052, p = 0.012, high, β = 0.055, p = 0.010) and happiness score before the pandemic (β = 0.467, p < 0.001), and satisfaction toward the measures against the COVID-19 at partners’ workplace (average, β = 0.129, p < 0.001; high, β = 0.279, p < 0.001), preventive behavior against COVID-19 (average, β = 0.055, p = 0.002; high, β = 0.045, p = 0.015) and positive attitudes/thinking (β = 0.087, p < 0.001) during the pandemic. In contrast, poor mental health (K6 ≥5, β = −0.042, p = 0.011) before the pandemic and negative changes during the pandemic (≥3, β = −0.085, p < 0.001) were negatively related to maternal happiness during the pandemic. Our final model explained 44.9% of the variance in mothers’ happiness during the COVID-19 pandemic.

**Conclusions:**

Satisfaction toward the measures against the COVID-19 at partners’ workplace, preventive behavior, and positive attitudes/thinking were especially important for maternal happiness during the COVID-19 pandemic. Future study is needed to consider measures against infectious diseases in the workplace that are desirable for the well-being of parents with young children, taking into account the gender perspective.

**Supplementary information:**

The online version contains supplementary material available at https://doi.org/10.1265/ehpm.22-00008.

## Background

The coronavirus disease 2019 (COVID-19) pandemic has had enormous impacts on the economy, health, human relationships, and daily life. During emergency situations, expectations of women to fulfill their roles may increase, which could in turn affect women’s well-being. For example, women are more likely to be the primary caregivers for their family, to experience a reduction in working hours, to be victims of violence, and to be exposed to a greater risk of infection through work and caregiving activities [1]. In addition, since living with young children was one of risk factors for mental health during the COVID-19 pandemic [2] and maternal mental health also affects their children [3, 4], this topic deserves more research attention.

In Japan, the Ministry of Education, Culture, Sports, Science and Technology [5] required schools to be temporarily closed from March 2nd, 2020, and the Head of the Novel Coronavirus Response Headquarters declared a national state of emergency on April 7th, 2020 [6]. However, the Japanese government did not implement compulsory measures such as lockdowns, and instead requested citizens to avoid closed spaces, crowded spaces, and close-contact, and to stay at home and refrain from traveling to other prefectures, without enforcing penalties for doing so. While the policies to combat the spread of COVID-19 were different between prefectures, most eating and drinking establishments were asked to reduce their opening hours, public spaces (e.g. libraries) were temporary closed, and public events including child medical check-ups were postponed in areas with high infection rates. Therefore, from March to May 2020, mothers in Japan may have faced challenging experiences. In addition, although most schools were reopened by June 2020, normal schooling was difficult in practice, and some kindergartens and other childcare facilities remained closed or reopened with reduced hours, asking parents to bring their children in only when absolutely necessary.

Previously, we focused on this period and conducted a baseline survey and a follow-up survey (in February and in June, 2020), and found that negative changes in circumstances/perceptions during the COVID-19 pandemic were related to the onset of depressive and anxiety symptoms in mothers of infants and/or preschoolers in Japan [7]. However, some women in that study exhibited improvements in depressive and anxiety symptoms, despite the burdens related to child care. This indicates that some mothers successfully coped with the challenging situations resulting from the COVID-19 pandemic.

Previous studies conducted in China have reported that specific precautionary measures against the spread of COVID-19 (e.g. wearing a mask and washing hands) were related to lower levels of depression, anxiety, and stress [8]. In addition, Guo et al [9] found that practical coping behaviors (e.g. “telling myself that everything will be better soon”, “wearing a mask when going outside”) were negatively associated with mental health problems during the COVID-19 pandemic. Although the terminology used differed slightly between these studies, preventive behaviors against COVID-19 and positive attitudes/thinking can be considered as one of the copings, which seemed essential to mental health during the COVID-19 [9].

In addition, our study revealed that increased partner’s time spent at home during COVID-19 were negatively related to the onset of depressive and anxiety symptoms among mothers [7], and this may imply that the measures against the COVID-19 at their partners’ workplace (e.g. flexibility in working style) may be an important factor for mothers’ mental health.

Various studies reported the associations between mental health and happiness [10–12], which can be regarded as a condition of psychological balance and harmony [13], and may help to prevent negative physiological outcomes in response to life events [14]. The definition of overall happiness is “the degree to which an individual judges the overall quality of his/her own life-as-a-whole favorably,” and this is an umbrella term for all that is good, and can be interchangeably used with ‘well-being’ or ‘quality of life’ [15] and linked with perceived social support [16]. In addition, happiness is important for both mothers and children. For example, researchers have reported that mothers with lower happiness levels are more likely to experience pregnancy-related risks, such as neonatal and infant death [17]; moreover, positive associations of happiness with children’s cognitive-executive functions and facial emotional recognition have been reported in mothers of children with Down syndrome [18]. Furthermore, Barak [19] reviewed articles related to the immune system and happiness, and reported that there are positive associations between happiness and optimism, mood, the number of T helper cells, and natural killer cell cytotoxicity. Therefore, to protect health among vulnerable populations such as mothers and children during the COVID-19 pandemic, maternal happiness should be paid attention to. Nonetheless, although the significant relationships between increasing COVID-19 cases and decreasing happiness have been reported worldwide [20], limited studies have focused on happiness during the pandemic [21, 22], and longitudinal studies related to maternal happiness are still lacking.

The aim of this study was to investigate the predictors for happiness in mothers of infants and/or preschoolers in Japan, using data obtained before and during the COVID-19 pandemic. In particular, we focused on the relationships between happiness and the possible factors, which might be especially observed in the COVID-19 pandemic.

## Method

### Participants and procedure

We used same dataset of the previous study [7]. In February 2020, we commissioned an Internet research company to conduct a baseline survey of 4,700 mothers with infants and/or preschoolers nationwide (100 mothers in each of 47 prefectures). The company partnered with several companies’ panels (ordinary citizens who registered of their own free will), and as of January 2020, approximately 4.73 million people had registered. The registered survey monitors were in their 20s (23%), 30s (23%), and 40s (22%); 62% were female, 59% were married, and 49% had children. The eligibility for our baseline study was women aged 20–49 years raising infants and/or preschoolers aged 0–6 years. Women who were raising more than one preschooler were asked to answer questions about their youngest child. The survey monitors were recruited through banner advertisements on the Internet, and no special privileges were given upon registration. Four months later, in June 2020, we commissioned the same research company to invite the women who had participated in the baseline survey to participate in a follow-up survey, and obtained responses from 2,489 subjects (response rate 53%).

### Measurements

#### Happiness

Although various measures of happiness have been developed, we used a single item that is commonly used [15], and its concurrent validity, convergent validity, and divergent validity were confirmed by the positive correlation with other happiness scale and related scales including satisfaction with life scale, optimism and hope, and the negative correlation with anxiety, pessimism, and insomnia [23]. Given that the present study compared happiness between two points in time, we used the following single question: “How happy are you now?” Participants were asked to rank their happiness on a scale ranging from 0 to 10 (0 = extremely unhappy, to 5 = neutral, to 10 = extremely happy) at both the baseline and the follow-up survey. This question has been widely used in Japan, including the Japanese government’s National Survey on Living Preferences [24], the local government survey and other studies [25].

#### Negative changes due to the COVID-19 pandemic

Negative changes related to the COVID-19 pandemic, a risk factor for mental health revealed in the previous study [7], were also used in this study. In detail, we assessed COVID-19 pandemic-associated negative changes between March to May in 2020 at the follow-up survey using items such as “Living in areas under special precautions”, “Increased financial difficulty”, “Fear of COVID-19 transmission”, “A shortage of relaxation time”, “Increased aggression from partner”, and “Increased sense of unfairness”. Items were scored as either “Yes” (1) or “No” (0). In addition, the item “Increased difficulty in child rearing” was rated on a four-point Likert scale (1 = agree, 2 = mostly agree, 3 = mostly disagree, and 4 = disagree), and scores were then dichotomized as “1” (for responses of 1 and 2), and “0” (for responses of 3 and 4). Participants were divided into four groups according to the total of these 7 items (0, 1, 2, and ≥3) [7].

#### Satisfaction toward the measures against the COVID-19 at partners’ workplace

As already mentioned, the measures against the COVID-19 at their partners’ workplace seemed to be an important factor for mothers’ well-being, and it may be reflected in satisfaction toward the partner’s workplace. To assess this, we used the following single question: “Are you satisfied with the measures against the COVID-19 at their partners’ workplace?”. Participants were asked to rank their satisfaction on a scale ranging from 0 to 10 (0 = not satisfied at all, to 5 = neutral, to 10 = very satisfied), which were divided into three groups according to tertiles (a score of 0–3 was recoded as “0”, a score of 4–5 was recoded as “1”, a score of 6 or more was recoded as “2”).

#### Coping behavior: preventive behavior against COVID-19 and positive attitudes/thinking

To assess preventive behavior against COVID-19, we asked the participants at the follow-up survey, “What did you do during the peak of the novel coronavirus pandemic between March and May, 2020?” Then, the following 8 items related to preventive behavior against the spread of COVID-19 were presented: “I thoroughly washed my own and my child’s/children’s hands after returning home (washing hands)”, “We wore face masks (wearing face masks)”, “I did not take my child/children to crowded places (avoiding crowded places)”, “I kept myself and my children at a distance from people to avoid infection (keeping social distance)”, “I refrained from going out (refrain from going out)”, “I paid attention to indoor ventilation (paying attention to ventilation)”, “I made sure my child/children ate a balanced diet (providing balanced meals)”, and “I was more careful than usual about changes in my child’s/children’s physical condition (taking care of child’s condition)”. For each item, participants responded with “Yes” (1) or “No” (0). The total of the 8 items ranged from 0–8, and participants were divided into three groups according to tertiles (scores of 0 to 5 were recorded as “0” (poor), scores of 6 and 7 were recoded as “1” (average), and a score of 8 was recoded as “2” (perfect)).

Positive attitudes/thinking were assessed by four items. Participants were asked about their experiences between March and May 2020 at the follow-up survey using the 4 following items: “In our family, we could talk with each other more often” (increasing conversation), “I tried to use my time at home productively” (using time productively), “I tried to think positively” (thinking positively), and “I think I did my best in the prevention of contracting and spreading COVID-19” (appraising one’s own efforts). The responses to these items were either “Yes” (1) or “No” (0). The total of the 4 items (ranged 0–4) was used as continuous variable.

#### Sociodemographic variables

Following to previous study [7], we used sociodemographic variables at the baseline, such as the participants’ age (20–29, 30–39, 40–49 years), annual household income in yen (<4,000,000; 4,000,000–5,999,999; ≥6,000,000; do not want to answer), educational background (junior high/high school, junior college/vocational school, university/postgraduate), employment status (employed full-time, employed but on child care leave, non-full time/self-employed, homemaker), marital status (married/had a partner, widowed/divorced/never married), number of children in their families (1, 2, ≥3), and child’s age (0–1, 2–3, ≥4 years). If participants had multiple children, they were requested to respond to the age and other child-related survey questions with regard to their youngest child.

#### Social support

To assess the number of social support resources available, we asked participants at the baseline, “If you have difficulties related to child rearing, what support can you obtain?” [26, 27]. Participants could choose from multiple responses, such as “husbands”, “parents”, “parents-in-law”, “friends/colleagues”, “neighbors”, “doctors”, “nurses/midwives”, “public nurses”, “teachers”, “public services”, “private services”, and “the Internet”. The total social support score (range 0–11) was calculated and participants were divided into three groups of available support resources (a score of 0–1 was recoded as “0”, a score of 2–3 was recoded as “1”, a score of 4 or more was recoded as “2”).

#### Mental health

We assessed mental health at the baseline by using the Kessler Psychological Distress Scale (K6) [28, 29]. Responses are graded on a five-point Likert scale (0 to 4), and higher scores of K6 indicate more severe psychological distress [28, 29]. The Japanese version of the K6 was shown to have screening performance for mood and anxiety disorders (cutoff values of 4/5 for mood/anxiety disorders and 12/13 for serious mental illness) [30], and we used a cutoff point of 4/5 to assess possibility of mood/anxiety disorders (Cronbach’s alpha = 0.92, scores range: 0–24, mean = 5.1 (SD = 5.3).

### Statistical analysis

Paired-samples t-test for happiness (baseline-follow-up surveys) was performed. To compare baseline data of participation and non-participation at follow-up survey, a Chi-squared test was performed. In addition, to show the characteristics of the participants and their happiness score, T-test or one-way analysis of variance were performed. For continuous variables, correlation coefficients with the happiness score were calculated. Then, hierarchical multiple regression analysis was performed using happiness as the dependent variable. First, sociodemographic variables and considerable predictors of happiness at the baseline were included in Model 1. Model 2 added numbers of negative changes related to COVID-19; Model 3 added satisfaction toward the measures against the COVID-19 at partners’ workplace; Model 4 added coping behavior (preventive behavior and positive attitudes/thinking). There were no missing data. STATA V15 (Stata Corp, College Station, TX, USA) was used for all statistical analyses, and a p-value of 0.05 was set as the significance level.

### Ethical considerations

An ethical review board at St. Marianna University School of Medicine approved this study (ID:4648). At the beginning of the online survey, participants provided informed consent. In addition, participants were informed that they could withdraw from the study at any time.

## Results

A total of 4,700 participants participated the baseline survey; of those, 2,489 participated in the follow-up survey (a response rate of 53.0%). The mean happiness score at baseline was 6.7 (SD ± 2.2), and at follow-up was 6.2 (SD ± 2.4), which was significantly decreased (p < 0.001) (Table 1).

**Table 1:** Happiness scores of two surveys

	**Baseline (n = 2,489)**	**Follow-up (n = 2,489)**	***P***
mean	6.7 (SD ± 2.2)	6.2 (SD ± 2.4)	<0.001^1)^

^1)^Paired-samples t-test

The mean age of participants was 35.3 years (SD ± 5.5, range 20–49 years), and the mean age of their children was 2.5 years (SD ± 2.0, range 0–6 years). Characteristics of the participants and their happiness scores at the follow-up survey are shown in Table 2. There were differences in happiness scores among most of the variables except for the mother’s age.

**Table 2:**
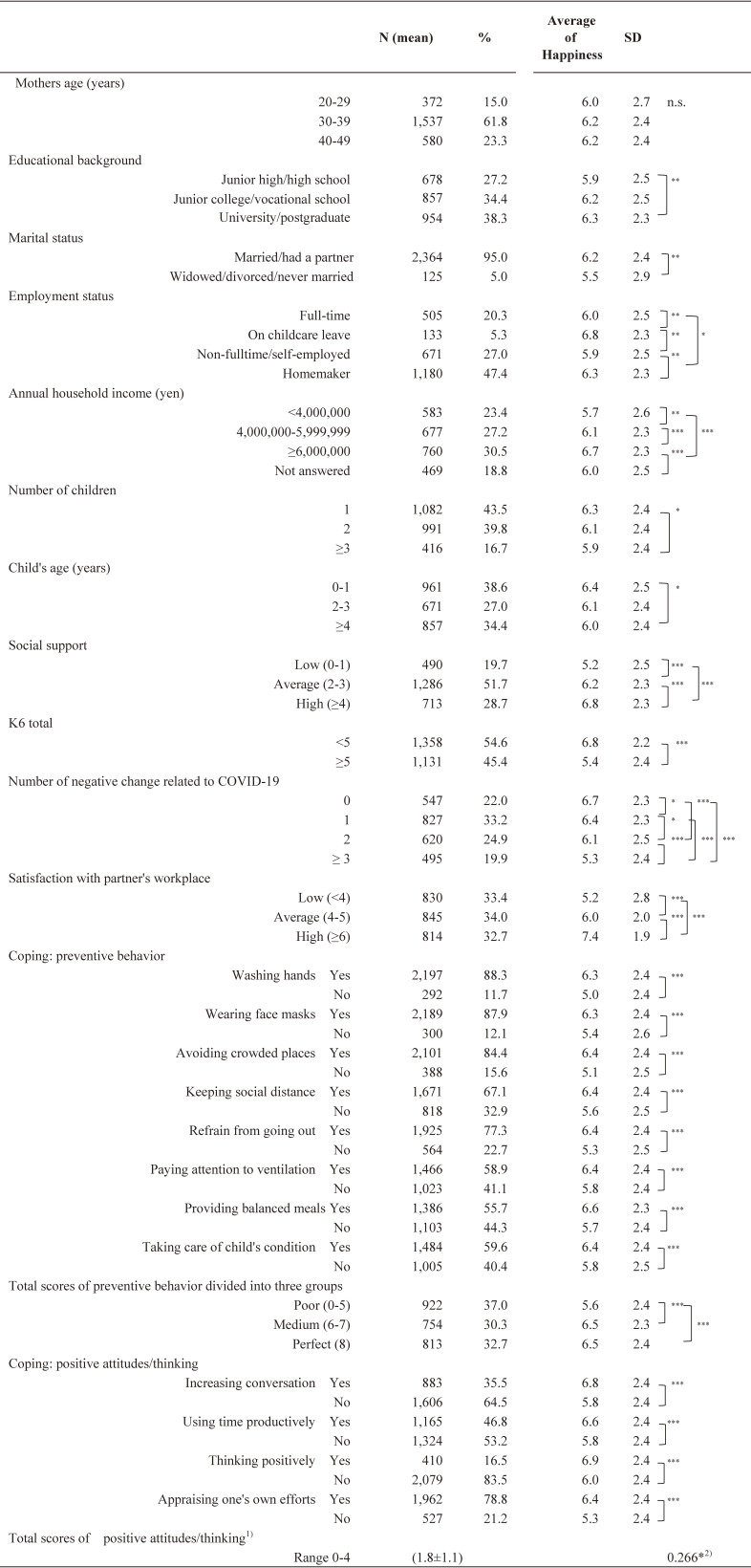
Characteristics of the participants and their happiness scores at the follow-up survey (n = 2,489)

^1)^This variable was treated as a continuous variable.

^2)^Pearson’s product-moment correlation coefficient. For the other variables, t-test or one-way analysis of variance were performed.

SD: Standard deviation

K6: The Kessler Psychological Distress Scale

^*^P < 0.05, ^**^P < 0.01, ^***^P < 0.001

Mean scores of happiness by employment status at baseline were 6.5 (SD ± 2.3) for full-time workers, 7.6 (SD ± 2.0) for those on childcare leave, 6.5 (SD ± 2.3) for non-full-time workers/self-employed and 6.8 (SD ± 2.2) for homemakers.

The comparison of continuous participants and dropouts in the follow-up survey are presented in Supplemental material 1. Mothers who were younger, employed, happier, had a lower annual income, less education, a younger child, and available support were more likely to drop out of the follow-up study.

Table 3 shows the result of hierarchical multiple regression analysis.

**Table 3:** Hierarchical multiple regression analysis predicting maternal happiness during the COVID-19

**Variables**	**Model 1**				**Model 2**				**Model 3**				**Model 4**			
			
	***β***	**p-value**	***R* ^2^**	**Adjusted ** ***R* ^2^**	***β***	**p-value**	***R* ^2^**	**Adjusted ** ***R* ^2^**	***β***	**p-value**	***R* ^2^**	**Adjusted ** ***R* ^2^**	***β***	**p-value**	***R* ^2^**	**Adjusted ** ***R* ^2^**
				
**Age (years)**			0.375	0.370			0.383	0.377			0.438	0.433			0.449	0.443
20–29	ref				ref				ref				ref			
30–39	0.026	0.276			0.030	0.199			0.025	0.267			0.026	0.243		
40–49	0.049	0.048			0.051	0.040			0.040	0.086			0.039	0.092		
**Educational background**																
University/postgraduate	ref				ref				ref				ref			
Junior high/high school	−0.017	0.364			−0.021	0.266			−0.011	0.541			−0.002	0.931		
Junior college/vocational school	0.004	0.830			0.001	0.963			0.008	0.636			0.013	0.467		
**Marital status**																
Widowed/divorced/never married	ref				ref				ref				ref			
Married/had a partner	−0.149	0.379			−0.014	0.404			−0.035	0.032			−0.030	0.064		
**Employment status**																
Full-time	ref				ref				ref				ref			
On childcare leave	0.013	0.480			0.015	0.400			0.014	0.409			0.010	0.550		
Non-fulltime/self-employed	0.014	0.511			0.020	0.351			0.009	0.666			0.000	0.992		
Homemaker	0.061	0.006			0.070	0.002			0.065	0.002			0.052	0.014		
**Annual household income (yen)**																
<4,000,000	ref				ref				ref				ref			
4,000,000–5,999,999	0.009	0.662			0.005	0.795			−0.005	0.784			−0.003	0.895		
≥6,000,000	0.065	0.003			0.065	0.003			0.040	0.054			0.035	0.089		
Not answered	−0.019	0.324			−0.025	0.204			−0.027	0.155			−0.020	0.278		
**Number of children**																
1	ref				ref				ref				ref			
2	−0.027	0.128			−0.023	0.191			−0.020	0.236			−0.029	0.084		
≥3	−0.022	0.221			−0.018	0.299			−0.013	0.450			−0.023	0.166		
**Child’s age (years)**																
0–1	ref				ref				ref				ref			
2–3	0.002	0.910			0.003	0.885			0.002	0.921			−0.003	0.844		
≥4	0.011	0.591			0.011	0.582			0.006	0.759			−0.001	0.975		

**Social support**																
Low	ref				ref				ref				ref			
Average	0.075	0.001			0.073	0.001			0.065	0.001			0.052	0.012		
High	0.095	<0.001			0.097	<0.001			0.076	<0.001			0.055	0.010		
**K6 total**																
<5	ref				ref				ref				ref			
≥5	−0.057	0.001			−0.044	0.013			−0.044	0.010			−0.042	0.011		
**Happiness(baseline)**	0.546	<0.001			0.535	<0.001			0.496	<0.001			0.467	<0.001		
**Number of negative change related to COVID-19**																
0					ref				ref				ref			
1					−0.016	0.432			0.001	0.958			−0.003	0.881		
2					−0.052	0.010			−0.026	0.183			−0.036	0.063		
≥3					−0.100	<0.001			−0.068	<0.001			−0.085	<0.001		
**Satisfaction toward partner’s workplace**																
Low									ref				ref			
Average									0.120	<0.001			0.129	<0.001		
High									0.283	<0.001			0.279	<0.001		
**Coping: preventive behavior**																
Poor													ref			
Average													0.055	0.002		
Perfect													0.045	0.015		
**Coping: positive attitudes/thinking**													0.087	<0.001		

K6: Kessler Psychological Distress Scale.

Sociodemographic variables and considerable predictors of happiness at the baseline were included in the Model 1. Model 2 added number of negative change related to COVID-19 to Model 1, Model 3 added satisfaction toward partner’s workplace to Model 2, and Model 4 added coping (preventive behavior and positive attitudes/thinking) to Model 3.

The adjusted R^2^ coefficients increased from model 1 (0.370) to model 4 (0.443). The final model (model 4) indicated that maternal happiness during COVID-19 pandemic was positively related to employment status (homemaker, β = 0.052, p = 0.014), levels of available social support (average, β = 0.052, p = 0.012, high, β = 0.055, p = 0.010) and happiness score before the pandemic (β = 0.467, p < 0.001), and satisfaction toward the measures against the COVID-19 at partners’ workplace (average, β = 0.129, p < 0.001; high, β = 0.279, p < 0.001), preventive behavior against COVID-19 (average, β = 0.055, p = 0.002; perfect, β = 0.045, p = 0.015) and positive attitudes/thinking (β = 0.087, p < 0.001) during the pandemic. In contrast, poor mental health (K6 ≥5, β = −0.042, p = 0.011) before the pandemic and experienced negative changes during the pandemic (≥3, β = −0.085, p < 0.001) were negatively related to maternal happiness during the pandemic. Our final model explained 44.9% of the variance in mothers’ happiness during the COVID-19 pandemic.

## Discussion

To the best of our knowledge, this is the first longitudinal study to identify the factors associated with happiness during the COVID-19 pandemic among mothers of infants and/or preschoolers. In this study, satisfaction toward the measures against the COVID-19 at their partners’ workplace was strongly related to happiness. As already mentioned, increased partner’s time spent at home during COVID-19 could be considered as protective factors of maternal mental health, but majorities of their partners could not increase the time they spent at home in Japan [7]. If the workplace allows flexible working or telecommuting, the partner may have spent more time at home, which may lead to the mother’s satisfaction with her partner’s workplace and increase her happiness.

To protect children against COVID-19, participants of this study tried to wash hands, avoid crowded places, observe social distancing, have a balanced diet, and so on. Such behaviors were observed in many mothers, and 32.7% of mothers were perfect in implementing all eight preventive behaviors, which strongly related to happiness. This result supports previous findings that preventive coping behavior is negatively associated with poor mental health [8, 9]. However, implementation of preventive behaviors in our study might be higher than other countries such as China (59.8% of respondents always wore a mask) [8], and the US (approximately 41% of the sample wore a mask before any mandates) [31]. In comparison, 87.9% of participants in this study wore face masks without any mandates; thus, such behaviors might be partially related to Japanese culture. For example, most public schools (elementary and junior high schools) in Japan have provided lunch, and students serving school lunches have been required to wear masks [32]; this may reduce students’ hesitation to wear masks when they reach adulthood.

Positive attitudes/thinking were positively associated with happiness, and perceiving increased conversation, using time productively, thinking positively and appraising one’s own efforts could be considered as effective ways to being happy during the COVID-19 pandemic. We considered that if participants feel that they did their best, their sense of control and positive appraisal of oneself might be increased, which might in turn increase their happiness. Therefore, it is important to praise the mothers who have been implementing the preventive behaviors of COVID-19, rather than blaming them for not being able to implement them. It would also be necessary for the mothers themselves to take a positive view of their own efforts, so that they can focus on what is possible during the COVID-19 pandemic.

On the contrary, mothers who experienced 3 or more negative changes during COVID-19 were less likely to be happy, which was similar to the results of the previous study focused on the onset of depressive and anxiety symptoms among mothers of infant/preschoolers [7]. Thus, experiencing 3 or more negative changes may affect maternal well-being, and especially need to pay attention to mothers’ such experiences (e.g. experiencing a shortage of relaxation time, increased difficulty in child-rearing, increased partner aggression and an increased sense of unfairness and so on).

The positive associations between social support and happiness have been consistently reported [16], which was also shown in our study. If participants had difficulties going out during the COVID-19 pandemic, mothers who had more sources of support may have found it easier to collect information, ask for help with child care and domestic duties, and have more opportunities to consult with health professionals than mothers who had less sources of support. Therefore, providing information, including various types of social support, may be one effective strategy to maintain/increase mothers’ happiness during pandemics.

In our study, mothers who were homemakers at the baseline were more likely to be happy than working mothers before and during the pandemic. This result is consistent with a previous study, which reported that homemakers with children have a higher score of happiness than working mothers [33]. Working mothers may have various difficulties continuing their works, or some of them had to leave their works to take care of children [34]. On the other hand, mothers who were homemakers might not face such dilemma and may have been more comfortable with spending time at home.

Happiness at the baseline predicted happiness during COVID-19, and this trend was also observed mental health before and during the COVID-19 pandemic [2]. Since the follow-up survey was conducted just four months after the baseline survey, it is quite natural that there is a strong correlation between the two surveys of happiness. Similarly, mothers who were poorer mental health before the pandemic were more likely to be unhappy during the pandemic. Since mental illness could be considered as one of the main causes of unhappiness [10], it is not surprising that poor mental health is strongly associated with poor happiness. Because the mental health and well-being of mothers affect their children and family members, it may be necessary to create a supportive environment in which mothers can remain stable, and learn how to be healthy during the pandemic.

The characteristics of mothers who dropped out of the follow-up study were younger (both mother and child), employed, had a lower annual income, less education, higher happiness scores, and a variety of social support. However, mental health was almost the same in both groups. Younger mothers may have younger children, and it is possible that they had placed their children in day-care centers and began working by the time of the follow-up survey. Such lifestyle changes might have reduced the time and motivation to participate in the follow-up survey. In addition, previous studies reported that participants who were younger age, lower annual income and less educated were more likely to drop out from longitudinal survey [35, 36], which were similar to our results. However, it is necessary to confirm whether the dropouts in the next survey would have the same characteristics as those in this survey.

## Limitations

This study has some limitations. The study was conducted by recruiting mothers registered with an Internet survey company, and the follow-up survey had a 53% response rate, these may have resulted in selection bias. In addition, although there was no difference in mental health, there were some trends, such as mothers with higher happiness scores dropped out, which may have affected the results of this study. Moreover, 53% of the mothers had some kind of job, and their satisfaction toward the COVID-19 measures at their own workplace may affect their happiness more strongly than satisfaction toward the COVID-19 measures at their partners’ workplace. However, this study was unable to examine the relationship between mothers’ satisfaction with their workplace and happiness. Furthermore, we were unable to clarify details such as what the covid-19 measures were taken in the partner’s workplace and which of these measures were related to the mother’s happiness. These were the serious limitations of the study. Therefore, to create a supportive environment, further research is needed to investigate what kind of COVID-19 measures at workplaces both fathers and mothers would be expected to increase/maintain well-being among parents of infants and/or preschoolers.

## Conclusion

This study found that satisfaction toward the measures against the COVID-19 at partners’ workplace, preventive behavior, and positive thinking/attitudes were especially important for maternal happiness during the COVID-19 pandemic. Future study is needed to investigate what COVID-19 measures are effective for the well-being of both mothers and fathers with young children, taking into account the gender perspective.

## Supplementary information

**Additional file 1:** **Supplemental material 1.** Comparison of continuous respondents and dropouts in the follow-up survey.

## Declarations

### Availability of data and materials

No data and materials are available.
