# Supplementary material for: Predictors of happiness during the COVID-19 pandemic in mothers of infants and/or preschoolers: a pre-COVID-19 comparative study in Japan
Source: Environ Health Prev Med. 2022 Mar 26;27:14. doi: 10.1265/ehpm.22-00008 (PMC9251627; doi:10.1265/ehpm.22-00008)
Supplement: Supplementary file 1 — Additional file 1: Supplemental material 1. Comparison of continuous respondents and dropouts in the follow-up survey. [file ehpm-27-014-s001.docx]

Supplemental material 1. Comparison of continuous respondents and dropouts in the follow-up survey

|  |  | Respondents  (n=2,489) | |  | Dropouts  (n=2,211) | |  |
| --- | --- | --- | --- | --- | --- | --- | --- |
|  |  | n | % |  | n | % | *p*-value |
| Mothers age (years) | |  |  |  |  |  |  |
|  | 20-29 | 372 | 14.9 |  | 547 | 24.8 | <0.001 |
|  | 30-39 | 1,537 | 61.8 |  | 1,348 | 61.0 |  |
|  | 40-49 | 580 | 23.3 |  | 315 | 14.2 |  |
| Educational background | |  |  |  |  |  |  |
|  | Junior high/high school | 678 | 27.2 |  | 764 | 34.6 | <0.001 |
|  | Junior college/vocational school | 857 | 34.4 |  | 734 | 33.2 |  |
|  | University/postgraduate | 954 | 38.3 |  | 713 | 32.2 |  |
| Marital status | |  |  |  |  |  |  |
|  | Married/had a~~s~~ partner | 2,364 | 95.0 |  | 2,073 | 93.8 | n.s. |
|  | Widowed/divorced/never married | 125 | 5.0 |  | 138 | 6.2 |  |
| Employment status | |  |  |  |  |  |  |
|  | Full-time | 505 | 20.3 |  | 468 | 21.2 | <0.001 |
|  | On childcare leave | 133 | 5.3 |  | 187 | 8.5 |  |
|  | Non-fulltime/self-employment | 671 | 27.0 |  | 612 | 27.7 |  |
|  | Homemaker | 1,180 | 47.4 |  | 944 | 42.7 |  |
| Annual household income (yen) | |  |  |  |  |  |  |
|  | <4,000,000 | 583 | 23.4 |  | 601 | 27.2 | <0.001 |
|  | 4,000,000-5,999,999 | 677 | 27.2 |  | 667 | 30.2 |  |
|  | ≥6,000,000 | 760 | 30.5 |  | 504 | 22.8 |  |
|  | Not answered | 469 | 18.8 |  | 439 | 19.9 |  |
| Number of children | |  |  |  |  |  |  |
|  | 1 | 1,082 | 43.5 |  | 946 | 42.8 | n.s |
|  | 2 | 991 | 39.8 |  | 857 | 38.8 |  |
|  | ≥3 | 416 | 16.7 |  | 408 | 18.5 |  |
| Child's age (years) | |  |  |  |  |  |  |
|  | 0-1 | 961 | 38.6 |  | 1,047 | 47.4 | <0.001 |
|  | 2-3 | 671 | 27.0 |  | 610 | 27.6 |  |
|  | ≥4 | 857 | 34.4 |  | 554 | 25.1 |  |
| Social support | |  |  |  |  |  |  |
|  | Low (0-1) | 490 | 19.7 |  | 359 | 16.2 | 0.007 |
|  | Average(2-3) | 1,286 | 51.7 |  | 1,169 | 52.9 |  |
|  | High (≥4) | 713 | 28.6 |  | 683 | 30.9 |  |
| K6 total |  |  |  |  |  |  |  |
|  | < 5 | 1,358 | 54.6 |  | 1,202 | 54.4 | n.s. |
|  | ≥5 | 1,131 | 45.4 |  | 1,009 | 45.6 |  |
| Happiness* Mean(±SD) | | 6.7 | (2.2) |  | 6.9 | (2.2) | <0.001 |
| * t-test was performed. For the other variables, Chi-square test was performed.  K6: Kessler Psychological Distress Scale. | | | | | | | |
